# Reversible Acute Kidney Injury Due to Suspected Paraneoplastic Glomerulopathy in Malignant Ovarian Germ Cell Tumor: A Case Report

**DOI:** 10.7759/cureus.83750

**Published:** 2025-05-08

**Authors:** Nozomi Furuzono, Shinichi Togami, Honami Oi, Aoi Iwao, Hiroaki Kobayashi

**Affiliations:** 1 Department of Obstetrics and Gynecology, Faculty of Medicine, Kagoshima University, Kagoshima, JPN

**Keywords:** acute kidney injury, case report, malignant ovarian germ cell tumor, paraneoplastic glomerular dysfunction, paraneoplastic glomerulopathy

## Abstract

Acute kidney injury (AKI) in patients with malignancies is typically caused by urinary obstruction, tumor infiltration, or metabolic derangements. However, paraneoplastic glomerulopathy remains an uncommon and under-recognized etiology of AKI, particularly in gynecologic malignancies. We describe the case of a 21-year-old woman who presented with abdominal distension and a large pelvic mass. Imaging and laboratory data suggested an International Federation of Gynecology and Obstetrics stage IIIC malignant ovarian germ cell tumor. Renal dysfunction was evident on admission and did not respond to intensive fluid resuscitation. The patient showed no signs of hydronephrosis, infection, or electrolyte abnormalities. Despite supportive care, her renal function continued to deteriorate, and continuous hemodiafiltration (CHDF) was initiated. Due to progressive clinical decline, emergency tumor debulking surgery was performed. Histopathology confirmed a mixed malignant ovarian germ cell tumor composed primarily of dysgerminoma and yolk sac tumor elements. Postoperatively, her renal function improved rapidly, and CHDF was discontinued by postoperative day 5. Full-dose bleomycin, etoposide, and cisplatin chemotherapy was commenced on postoperative day 17 and completed without dose reduction or major toxicity. Currently, the patient remains in complete remission. This case suggests the possibility of paraneoplastic glomerulopathy as a reversible cause of AKI in patients with ovarian germ cell tumors. The dramatic improvement in renal function after tumor resection supports this hypothesis. In this case, prompt surgical intervention not only resolved the renal impairment but also allowed for the timely administration of curative chemotherapy. To our knowledge, this is the first report to suggest an association between paraneoplastic glomerular dysfunction and malignant ovarian germ cell tumors.

## Introduction

Malignant ovarian germ cell tumors account for 3.2% of all malignant ovarian tumors, with mixed germ cell tumors being extremely rare, comprising only 0.3% of cases. Histologically, malignant ovarian germ cell tumors include dysgerminoma, yolk sac tumor, and immature teratoma, with mixed germ cell tumors encountered less frequently than the others [1]. One of the most notable characteristics of malignant ovarian germ cell tumors is their predominance in adolescents and young adults, typically occurring in their 10s and 20s. Due to their high sensitivity to chemotherapy, fertility-sparing surgery is often considered, regardless of the disease stage [2].

Acute kidney injury (AKI) associated with malignancy is typically caused by tumor-related urinary tract obstruction, direct tumor invasion of the kidneys, or humoral hypercalcemia of malignancy - resulting from tumor-derived parathyroid hormone-related protein, activated vitamin D, or extensive bone metastases leading to hypercalcemia [3]. However, there have been no prior reports of AKI complicating malignant ovarian germ cell tumors without these underlying mechanisms.

Herein, we describe the rare case of a patient with a mixed malignant ovarian germ cell tumor, complicated by AKI, which progressed to the point of requiring emergency renal replacement therapy (RRT).

## Case presentation

A 21-year-old woman (gravida 0) presented with a chief complaint of abdominal distention to a local gynecological clinic. Transabdominal ultrasonography revealed a pelvic tumor extending up to the umbilicus. Subsequently, she was referred to our department for further evaluation and treatment. Upon arrival, she was ambulatory, with a performance status of 0. The physical examination revealed a palpable tumor in the left lower abdomen, extending to one fingerbreadth above the umbilicus. The uterus was mobile and approximately the size of a hen’s egg, while the left adnexa was not palpable. On speculum examination, the cervix was deviated to the left, suggesting uterine displacement due to a right adnexal tumor.

Transvaginal ultrasonography revealed a solid pelvic mass with areas of high and low echogenicity, along with ascites and a 5-cm nodule in the pouch of Douglas, suggesting peritoneal dissemination. Laboratory test results indicated mild anemia (white blood cell count: 8,560/μL; hemoglobin level: 10.1 g/dL; platelet count: 427,000/μL) and impaired renal function (serum creatinine (Cre) level: 2.24 mg/dL; creatinine clearance (CCr): 30.73 mL/min). Tumor markers were significantly elevated: alpha-fetoprotein (AFP) level: 159 ng/mL (normal <10 ng/mL); cancer antigen 125 (CA125) level: 435 U/mL; carbohydrate antigen 19-9 level: 4.0 U/mL; and human chorionic gonadotropin (hCG) level: 670.3 IU/L (normal <5 IU/L in non-pregnant women). The level of lactate dehydrogenase (LDH), a tumor marker associated with dysgerminoma, was markedly elevated at 7,095 IU/L (Table 1).

**Table 1:** Laboratory test results

Parameter	Result	Reference Range
White blood cell count	8,560/μL	3,300-8,600/μL
Hemoglobin	10.1 g/dL	11.6-14.8 g/dL
Platelet count	427,000/μL	158,000-348,000/μL
Serum creatinine	2.24 mg/dL	0.46-0.79 mg/dL
Creatinine clearance	30.73 mL/min	>60 mL/min
Alpha-fetoprotein (AFP)	159 ng/mL	<7 ng/mL
Cancer antigen 125 (CA125)	435 U/mL	<35 U/mL
Carbohydrate antigen 19-9 (CA19-9)	4.0 U/mL	<37 U/mL
Human chorionic gonadotropin (hCG)	670.3 IU/L	<5 IU/L (non-pregnant women)
Lactate dehydrogenase (LDH)	7,095 IU/L	124-222 IU/L

**Figure 1:**
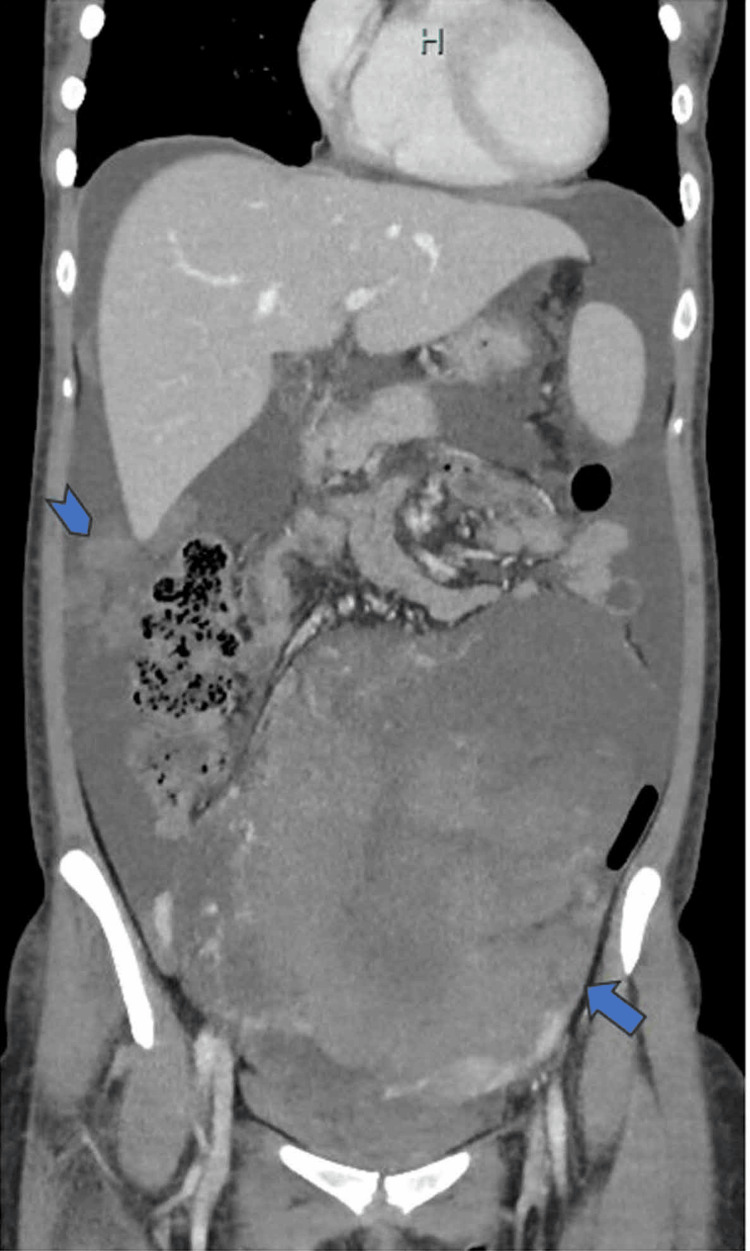
Computed tomography imaging A 24-cm right ovarian mass extending from the pelvic cavity to the upper abdomen is observed (arrow), along with scattered intraperitoneal dissemination (arrowhead).

Pelvic contrast-enhanced magnetic resonance imaging demonstrated a 24-cm solid tumor originating from the right adnexa, with low signal intensity on T1-weighted images and slightly high signal intensity on T2-weighted images, along with diffusion restriction. The uterus was displaced to the left by the tumor. Computed tomography showed no distant metastasis but revealed para-aortic lymphadenopathy, with a short axis of 10 mm, suggesting lymph node metastasis. Additionally, peritoneal dissemination, with nodules exceeding 2 cm in diameter, was identified in the upper abdomen (Figure 1).

We observed no hydronephrosis or urinary tract involvement. On the basis of these findings, we diagnosed the patient with suspected stage IIIC malignant ovarian germ cell tumor [4].

Due to the presence of renal dysfunction, urgent treatment was deemed necessary, and the patient was admitted two days after her initial visit to our department (day X+2). Upon admission, blood chemistry test results revealed a rapid progression of renal dysfunction over the preceding two days, with Cre increasing to 4.60 mg/dL and CCr decreasing to 14.96 mL/min. The patient’s urine output was 300 mL/day, which was below 0.5 mL/kg/hour. Thus, we diagnosed AKI on the basis of the Kidney Disease: Improving Global Outcomes (KDIGO) diagnostic criteria [5], which the patient met. No significant electrolyte abnormalities, including serum calcium levels, were observed, and transabdominal ultrasonography did not reveal bilateral hydronephrosis. Given the presence of abdominal distention, poor oral intake following a recent coronavirus disease infection, and a fractional excretion of sodium of 0.57% (below 1%), prerenal azotemia was suspected.

Despite consultation with nephrology and aggressive fluid resuscitation, renal function deteriorated further on the following day (day X+3), with the Cre level increasing to 6.54 mg/dL and CCr decreasing to 10.53 mL/min. The patient met the clinical indications for emergency RRT according to the KDIGO guidelines [5]. She was unresponsive to diuretics, with a 3-kg weight gain over two days. Although serum potassium levels remained within normal limits and no uremic symptoms were noted, arterial blood gas analysis revealed severe metabolic acidosis (pH: 7.345; bicarbonate level: 18.6 mmol/L; base excess: -7.3 mmol/L). Consequently, emergency RRT was initiated, and the patient was transferred to the intensive care unit, where continuous hemodiafiltration (CHDF) was initiated and led to improvements in fluid overload and metabolic acidosis. However, urine output remained minimal, and renal function showed no significant improvement.

**Figure 2:**
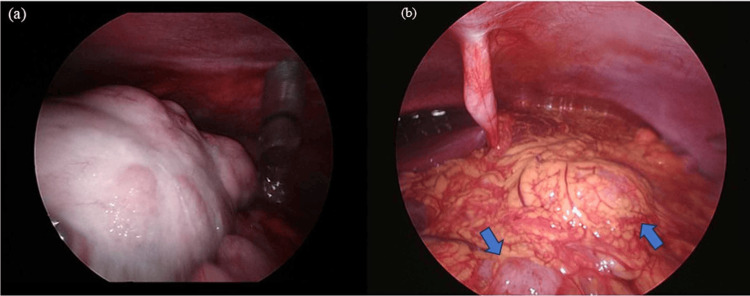
Findings of diagnostic laparoscopy (a) The tumor is occupying the pelvic cavity; (b) multiple peritoneal implants are observed on the omentum (arrows).

The etiology and pathophysiology of AKI remained unclear in this patient, but given the need for rapid intervention to improve the patient’s overall condition, diagnostic laparoscopic surgery was performed on day X+10 (Figure 2).

**Figure 3:**
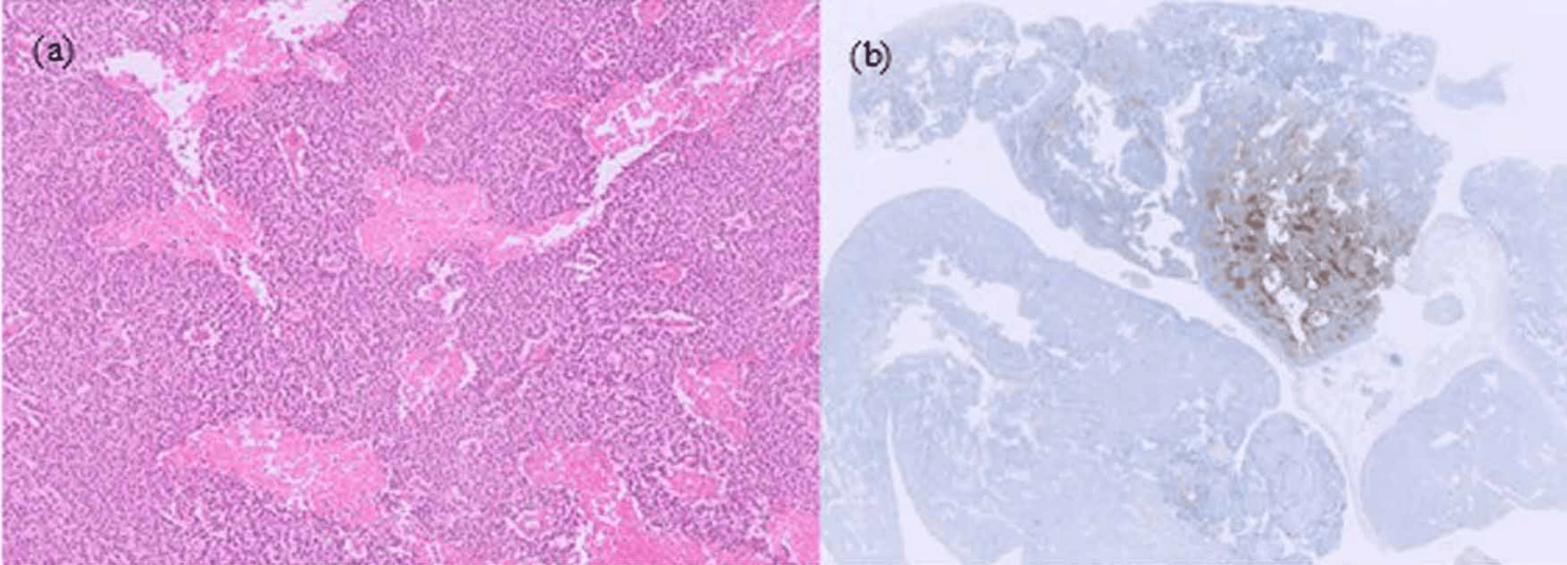
Histopathological findings of the resected specimen (a) Low-power view reveals nests with trabecular arrangements of polygonal tumor cells (hematoxylin and eosin staining); (b) low-power view reveals partial positivity of alpha-fetoprotein staining.

Intraoperative assessment using the Predictive Index [6] yielded a score of 4, indicating feasibility for primary debulking surgery. Thus, the procedure was converted to an open laparotomy, and right salpingo-oophorectomy, partial omentectomy, appendectomy, and resection of the disseminated nodules were performed. Macroscopic residual tumors included a 5-cm nodule in the pouch of Douglas and a 1-cm nodule around the ileocecal region, with a calculated reduction rate of 98%. The pathological diagnosis was mixed malignant ovarian germ cell tumor, consisting of undifferentiated germ cell tumor (95%) and yolk sac tumor (5%) (Figure 3).

Remarkably, by postoperative day 5 (day X+15), urine output had increased to >30 mL/hour, the Cre level improved to 2.8 mg/dL, and CCr increased to 24.59 mL/min, meeting the KDIGO criteria for discontinuation of CHDF [5]. While early initiation of postoperative bleomycin, etoposide, and cisplatin (BEP) as chemotherapy was considered, dose reduction (50%-75%) is recommended when CCr is <30 mL/min [7]. Given the need to administer full-dose chemotherapy, BEP initiation was postponed for one week to allow for further renal recovery.

**Figure 4:**
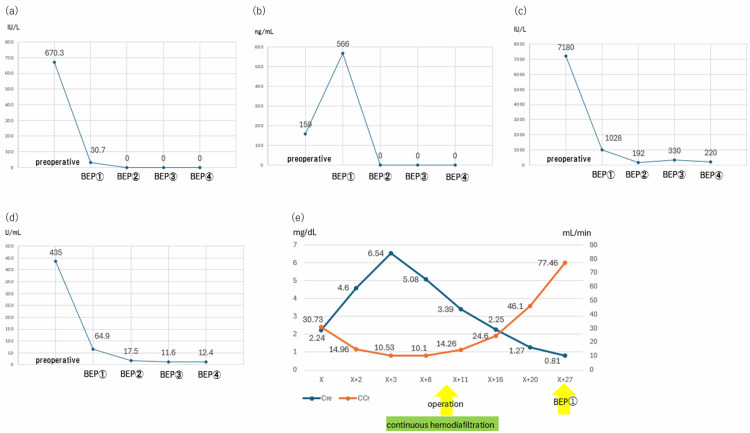
Trends in tumor markers and renal function (a) AFP (alpha-fetoprotein); (b) CA125 (cancer antigen 125); (c) LDH (lactate dehydrogenase); (d) hCG (human chorionic gonadotropin); (e) Renal function BEP, bleomycin, etoposide, and cisplatin

By postoperative day 17 (day X+27), renal function had normalized (Cre level: 0.81 mg/dL; CCr: 88.49 mL/min), allowing for the initiation of BEP chemotherapy without dose reduction. Serial monitoring of tumor markers (AFP, CA125, hCG, and LDH) following BEP chemotherapy is shown in Figures 4a-4e.

By the start of the third cycle of BEP, all tumor marker values had normalized, and imaging studies performed after completing four cycles of BEP confirmed a complete response. The patient has been followed up on an outpatient basis, without any evidence of recurrence to date.

## Discussion

The present case underscores the importance of considering tumor-related factors as potential contributors to AKI, even in the absence of common mechanisms such as urinary obstruction or hypercalcemia. Our patient initially had normal renal function but developed severe AKI that necessitated emergency RRT. Despite adequate fluid resuscitation, renal function continued to deteriorate, but it improved rapidly following tumor debulking surgery. Possible causes of AKI in malignant ovarian germ cell tumors include tumor lysis syndrome associated with treatment initiation [8] or drug-induced nephrotoxicity during BEP chemotherapy [7]. However, these mechanisms are treatment-related and were not applicable in this case. Causes of renal impairment associated with malignant tumors [9], including hematologic malignancies, are summarized in Table 2.

**Table 2:** Etiology and pathophysiology of cancer-related AKI AKI, acute kidney injury

Category	Mechanism
Direct mechanisms of the tumor	Direct infiltration of tumor cells into the kidney
	Urinary tract obstruction
	Compression of the renal vasculature
Indirect mechanisms of the tumor	Renal calcification
	Cast nephropathy due to multiple myeloma
	Immunological abnormalities (paraneoplastic glomerulopathy)
	Electrolyte imbalance
	Disseminated intravascular coagulation
	Urinary tract infections
Treatment induced	Tumor lysis syndrome
	Drug-induced nephrotoxicity
	Radiation nephropathy

Paraneoplastic glomerulopathy, a form of glomerular injury triggered by various hormones, growth factors, cytokines, and tumor-specific antigens produced by tumor cells, can occur independently of tumor invasion or metastasis [10]. Paraneoplastic glomerulopathy is most commonly associated with membranous nephropathy in solid tumors, particularly gastric, esophageal, renal, and lung cancers [11], and renal dysfunction secondary to paraneoplastic glomerulopathy typically improves or resolves with effective treatment of the primary malignancy. The diagnostic criteria for paraneoplastic glomerulopathy include: (1) exclusion of other apparent causes of renal dysfunction, (2) onset of renal dysfunction after the development of malignancy, and (3) improvement of renal function following successful treatment of the malignancy, with recurrence or worsening of renal dysfunction correlating with disease progression. Notably, renal biopsy is not mandatory for diagnosis [10].

Although there have been no previous reports of paraneoplastic glomerulopathy associated with malignant ovarian germ cell tumors [11], this diagnosis was strongly suspected in the present patient based on the following factors: (1) the absence of tumor infiltration or metastasis affecting the urinary tract, (2) lack of response to adequate fluid management, (3) absence of electrolyte disturbances, and (4) rapid improvement of renal function following tumor debulking surgery, which effectively treated the underlying malignancy. These findings suggest that paraneoplastic glomerulopathy was the most likely mechanism underlying the AKI observed in this patient.

BEP chemotherapy remains the standard adjuvant chemotherapy for malignant ovarian germ cell tumors and has demonstrated excellent treatment outcomes [1]. Maintaining the intensity of BEP chemotherapy, including adherence to the recommended dosage and treatment schedule, is crucial for achieving optimal outcomes [1]. Attempts to substitute cisplatin with carboplatin, due to concerns about nephrotoxicity and gastrointestinal toxicity in the treatment of testicular germ cell tumors, result in poorer prognoses [12]. In this case, the decision to delay BEP chemotherapy by a few days after surgery allowed for sufficient renal recovery, enabling the administration of full-dose BEP chemotherapy without the need for dose reduction. This approach contributed significantly to the successful completion of the treatment regimen and to the favorable outcome observed in this patient.

Although the diagnostic value of high-resolution ultrasound images and color Doppler evaluation is widely recognized in the characterization of adnexal masses, we regret that high-quality grayscale and Doppler images were not available for this particular case. This limitation precluded the inclusion of representative imaging figures in the manuscript.

Nonetheless, the lesion’s features were assessed based on standard sonographic parameters during the examination. In clinical practice, risk stratification tools such as the Risk of Malignancy Index (RMI), the International Ovarian Tumor Analysis (IOTA) Simple Rules, and the ADNEX (Assessment of Different NEoplasias in the adneXa) model are often employed to support the diagnosis. These models integrate factors such as menopausal status, CA-125 level, and specific ultrasound characteristics to estimate the probability of malignancy. Although these models were not formally applied in this case, their potential utility in similar clinical scenarios should be emphasized. Future studies should aim to incorporate such standardized models to enhance objectivity and reproducibility in the evaluation of adnexal lesions.

Moreover, this case report provides a detailed account of the patient's clinical presentation, physical findings, and initial sonographic features to support diagnostic reasoning in similar cases. It is important to note, however, that accurate interpretation of adnexal ultrasound findings requires experienced and well-trained personnel, as subtle features may be overlooked by less experienced examiners.

In many clinical settings, ultrasound serves as the first-line and often the most critical diagnostic tool. When interpreted correctly, it may offer sufficient information to reach a presumptive diagnosis without the need for additional imaging. This is particularly relevant in resource-limited environments, where access to advanced imaging modalities may be restricted. Acknowledging this, we emphasize the need for both comprehensive clinical evaluation and adequate operator training to ensure reliable interpretation and appropriate management.

Finally, recent advances in artificial intelligence (AI) have opened new possibilities in medical imaging analysis, including the evaluation of adnexal lesions. Although AI-based diagnostic tools were not applied in the present case, future studies may benefit from incorporating such technologies to improve diagnostic accuracy and reduce interpretation time. Integrating AI into ultrasound assessment may also help minimize inter-observer variability and support clinical decision-making, particularly in settings with limited access to expert operators.

## Conclusions

Prompt treatment of the primary malignancy in this patient led to the restoration of renal function, allowing for the successful administration of full-dose BEP chemotherapy without dose reduction. The ability to maintain the full treatment intensity of BEP chemotherapy was a critical factor contributing to the achievement of complete remission in this patient.
